# Electroacupuncture Alleviates Pain Responses and Inflammation in a Rat Model of Acute Gout Arthritis

**DOI:** 10.1155/2018/2598975

**Published:** 2018-03-19

**Authors:** Wenxin Chai, Yan Tai, Xiaomei Shao, Yi Liang, Guo-qing Zheng, Ping Wang, Jianqiao Fang, Boyi Liu

**Affiliations:** ^1^Department of Neurobiology and Acupuncture Research, The Third Clinical Medical College, Zhejiang Chinese Medical University, Hangzhou 310053, China; ^2^Department of Laboratory and Equipment Administration, Zhejiang Chinese Medical University, Hangzhou 310053, China; ^3^Department of Neurology, The Second Affiliated Hospital and Yuying Children's Hospital of Wenzhou Medical University, Wenzhou 325027, China; ^4^Department of Pathology, School of Basic Medical Science, Zhejiang Chinese Medical University, Hangzhou 310053, China

## Abstract

Acute gout arthritis is one of the most painful inflammatory conditions. Treatments for gout pain are limited to colchicine, nonsteroidal anti-inflammatory drugs, and corticosteroids, which oftentimes result in severe adverse effects. Electroacupuncture (EA) has been proved to be effective in relieving many inflammatory pain conditions with few side effects. Here, we aim to investigate the therapeutic potentials of EA on pain and inflammation of a rat model of acute gout arthritis and underlying mechanisms. We found that 2/100 Hz EA produced the most robust analgesic effect on mechanical hyperalgesia of acute gout arthritis rat model compared with 2 and 100 Hz. EA produced similar analgesic effect compared with indomethacin. 2/100 Hz EA also significantly alleviates the ongoing pain behavior, thermal hyperalgesia, and ankle edema. Locally applied *μ* and *κ*-opioid receptor antagonists but not adenosine A_1_ receptor antagonist significantly abolished the analgesic effect of EA. Locally applied *μ* and *κ*-opioid receptor agonists produced significant antiallodynia on acute gout arthritis rats, mimicking EA. Furthermore, 2/100 Hz EA upregulated *β*-endorphin expression in inflamed ankle skin tissue. Our results demonstrated, for the first time, that EA can be used for relieving acute gout arthritis with effect dependent on peripheral opioid system and comparable with the one obtained with indomethacin.

## 1. Introduction

Gout is recognized as one of the most acute painful symptoms that affect human beings [1, 2]. Gout is the most common inflammatory arthritis in older people, with an incidence of 1.4 in women and 4.0 in men per 1,000 persons [2–4]. Due to the aging population and changes of lifestyle, the incidence and prevalence of gout are steadily rising [3]. Acute gout arthritis is characterized with sudden occurrence, extremely painful, and inflammatory attacks to the joints. It is now generally accepted that the deposition and crystallization of monosodium urate (MSU) in and around the joint trigger the acute inflammation and initiate the acute gout attack [5]. Gout pain is undoubtedly one of the most serious pain symptoms and can severely affect the life quality of patients [2]. Therefore, the development of efficient analgesic methods for relieving gout pain is of high clinical significance. Current therapeutic methods for relieving gout pain are still limited to colchicine, nonsteroidal anti-inflammatory drugs (NSAIDs), and corticosteroids. Unfortunately, the usage of these drugs oftentimes leads to severe adverse effects, such as gastrointestinal adverse effects, chronic renal insufficiency, dysphoria, and immune suppression [1].

Electroacupuncture (EA) is a technique that integrates traditional acupuncture and modern electrotherapy. It can produce satisfactory analgesic effect on many acute and chronic pain conditions, whereas it exerts few side effects [6, 7]. Animal studies by us and others have shown that EA inhibits pain response in a variety of inflammatory pain models, including complete Freund's adjuvant- (CFA-) induced inflammatory pain, collagen-induced rheumatoid arthritis pain, postincisional pain, and fibromyalgia [8–12]. In clinic, it is reported that EA can improve the visual analogue scale score of acute gout arthritis patients [13–15]. However, until now, it is still not known whether EA can alleviate pain responses in animal models of acute gout arthritis and if so, what the underlying mechanisms could be.

It is well established that endogenous opioid and other nonopioid systems, such as adenosine, are important mechanisms underlying acupuncture-induced analgesia [16–18]. Early studies revealed a crucial role of central opioids in mediating acupuncture-induced analgesia [16, 18]. Subsequent studies also identified important roles of peripheral opioid and adenosine systems in acupuncture-induced analgesia [11, 12, 17, 19–24]. Here in the present study, we established a rat model of acute gout arthritis via MSU injection into ankle joint and tested whether EA could alleviate pain responses and inflammation in this model. We further explored which endogenous analgesic system mediated the effect of EA. Our study revealed that 2/100 Hz EA produced significant antiallodynia and anti-inflammatory effects on acute gout arthritis, which is comparable to the widely used NSAIDs indomethacin. The antiallodynia of EA is largely abolished by locally applied *μ*- and *κ*-opioid receptor antagonist. In turn, local application of *μ*- and *κ*-opioid receptor agonists exerts significant antiallodynia on acute gout arthritis, which mimics that of EA. Our findings suggest that EA can be a potential therapeutic option for acute gout arthritis.

## 2. Materials and Methods

### 2.1. Animals

*Male Sprague-Dawley *(SD)* rats* (from Laboratory of Animal Research Center, Zhejiang Chinese Medical University, Hangzhou, China), weighting 180–240 g, were used in this study. The rats were housed six per cage on a 12 h light/dark cycle with controlled temperature. Food and water were provided ad libitum. All rats were used strictly in accordance with the National Institutions of Health Guide for the Care and Use of Laboratory Animals.

### 2.2. MSU-Induced Acute Gout Arthritis Model Establishment

MSU was suspended in sterile phosphate buffered saline (PBS). 70% alcohol was used to disinfect the ankle area before the procedure. After making a small skin incision, 1.25 mg MSU (in 50 *μ*l volume) was injected into the rat ankle joint through a 21-gauge needle inserted just medial to the tendon of the tibialis anterior with its tip bevelled to 45° [25, 26]. Control group received injection of 50 *μ*l PBS. A successful gout arthritis model establishment was judged by obvious ankle swelling and mechanical hyperalgesia 4 or 6 h after the injection [25, 27].

### 2.3. EA Treatment

The procedure of EA was carried out according to our previous study [9]. Rats were loosely immobilized and four stainless steel acupuncture needles of 0.25 mm diameter were inserted at a depth of 5 mm into bilateral Zusanli (ST36, 5 mm lateral to the anterior tubercule of the tibia) and Kunlun (BL60, at the ankle joint level and between the tip of the external malleolus and calcaneus) acupoints. The needles were connected with HANS acupuncture point Nerve Stimulator (LH-202H). Square wave current output (pulse width: 0.2 ms) and intensities ranging 1-2 mA (each intensity for 15 min, a total of 30 min) were delivered for a period of 30 min. For Sham EA treatment, rats were inserted with needles subcutaneously into ST36 and BL60, but with no electrical discharge.

### 2.4. Determination of the Ankle Edema

Edema was observed as an increase in ankle diameter, as measured by a digital caliper, and was calculated as the difference between the basal value and the test value (observed at different time points after intra-articular (i.a.) injection of MSU or PBS) [27].

### 2.5. Ongoing Pain Score Evaluation

The ongoing pain score was evaluated as reported previously [25]. Briefly, rats were individually placed in transparent plexiglass chambers and habituated for 30 min. Each rat was observed for a standard period of 5 min. Under the chamber a mirror was set at a 45° angle to allow a clear view of the rats' feet. The amount of weight (paw pressure) the rat was willing to put on the hind paw of the injected limb was evaluated and categorized according to the following scale: 0 = normal paw pressure, equal weight on both hind paws; 1 = slightly reduced paw pressure (paw is completely on the floor but toes are not spread); 2 = moderately reduced paw pressure (foot curled with only some parts of the foot lightly touching the floor); 3 = severely reduced paw pressure (foot elevated completely) [25].

### 2.6. Determination of Mechanical Allodynia

Rats were individually placed in transparent plexiglass chambers on an elevated mesh floor and were habituated for 30 min before test. The mechanical allodynia was determined using a series of calibrated von Frey filaments (UGO Basile, Italy) applied perpendicularly to the midplantar surface of the hind paws, with sufficient force to bend the filament slightly for 3–5 s [28]. An abrupt withdrawal of the paw and licking and vigorously shaking in response to stimulation were considered pain-like responses. The threshold was determined using the up-down testing paradigm, and the 50% paw withdrawal threshold (PWT) was calculated by the nonparametric Dixon test [29, 30]. A baseline test of PWT was done every day for 3 consecutive days before the formal test to acclimatize the rats and ensure that there were no differences among groups.

### 2.7. Determination of Thermal Hyperalgesia

The Plantar Test Apparatus (Ugo Basile, Italy) was used to evaluate the hypersensitivity to heat stimulation (heat hyperalgesia). Rats were habituated for 30 min before the test. A radiant light beam generated by a light bulb was directed into the right hind paw in order to determine the paw withdrawal latency (the time spent to remove the paw from the stimulus). A 20 s cutoff threshold was set to avoid excessive heating to cause injury. Significant decreases in paw withdrawal latency were interpreted as heat hyperalgesia. All above behavior tests are conducted by an experimenter blinded to experimental conditions.

### 2.8. Histopathological Assessment of Ankle Joints

For microscopic evaluation of the ankle joint, rats were sacrificed 8 h after MSU injection. After the rats were sacrificed, the ankles were dissected. Periarticular tissues were removed. The ankles were fixed in 10% formalin and decalcified using 10% EDTA. They were then dehydrated by processing in different grades of alcohol/chloroform mixture and embedded in paraffin. The sections were cut in 6 *μ*m thickness and were stained with hematoxylin and eosin for pathology examination under the light microscope [31] (Leica, Germany).

### 2.9. Immunohistochemistry

Procedures were performed as described before [32, 33]. Briefly, ankle skin samples were collected and frozen in frozen tissue matrix (OCT) and then cut by cryostat in 14-*μ*m sections (Thermofisher, USA). The skin was postfixed with cold acetone for 10 min and air-dried before immunostaining. For immunostaining, the sections were first blocked with 1% BSA plus 10% goat serum for 2 h at room temperature. The sections were then incubated overnight at 4°C with primary antibody against *β*-endorphin (Abcam, Cambridge, UK) and corresponding secondary antibody (Invitrogen, Carlsbad, CA, USA). Nuclei were stained with DAPI (Abcam, Cambridge, UK). Fluorescence signals were detected by Nikon A1R laser scanning confocal microscope (Nikon, Japan). The fluorescence intensity of the stained area in each image was measured by ImageJ software and then averaged and analyzed.

### 2.10. Chemicals

MSU, naloxone, nor-binaltorphimine, KW-3902, DAMGO, and (±) U50488 were purchased from Tocris (Minneapolis, MN, USA); indomethacin was purchased from Sigma (St. Louis, MO, MO, USA).

### 2.11. Drug Treatment

Opioid receptor antagonists naloxone (40 *μ*g/ankle), nor-binaltorphimine (76 *μ*g/ankle), naltrindole (48.7 *μ*g/ankle), and adenosine A_1_R antagonist KW-3902 (600 *μ*g/ankle) were dissolved in PBS and injected intra-articularly (i.a.) into the ankle 30 min before EA treatment. The dosages of the antagonists chosen above are effective local dosage without systematic effect used in previous studies [12, 34, 35]. Opioid receptor agonist DAMGO (4.9 *μ*g/ankle) and (±) U50488 (1 *μ*g/ankle) were dissolved in PBS and injected (i.a.) into the ankle 30 min before PWT evaluation. The dosages of the agonists chosen above are effective local dosage used in previous studies [36, 37]. Indomethacin (5 mg/kg) was prepared in dimethyl sulfoxide (DMSO) as a stock, further diluted in PBS and applied through intraperitoneal (i.p.) injection [38]. Details of drug effects, dosage, and rout of application can be found in Table 1.

### 2.12. Statistics

One-way or two-way ANOVA followed by Tukey's post hoc test was used for comparison among groups. Comparison is considered significantly different if the *p* value is less than 0.05. Data in bar graphs are expressed as means ± SE.

## 3. Results

### 3.1. The Establishment of a Rat Model of MSU Crystal-Induced Acute Gout Arthritis and Evaluation of the Inflammation and Pain Properties

**Figure 1:**
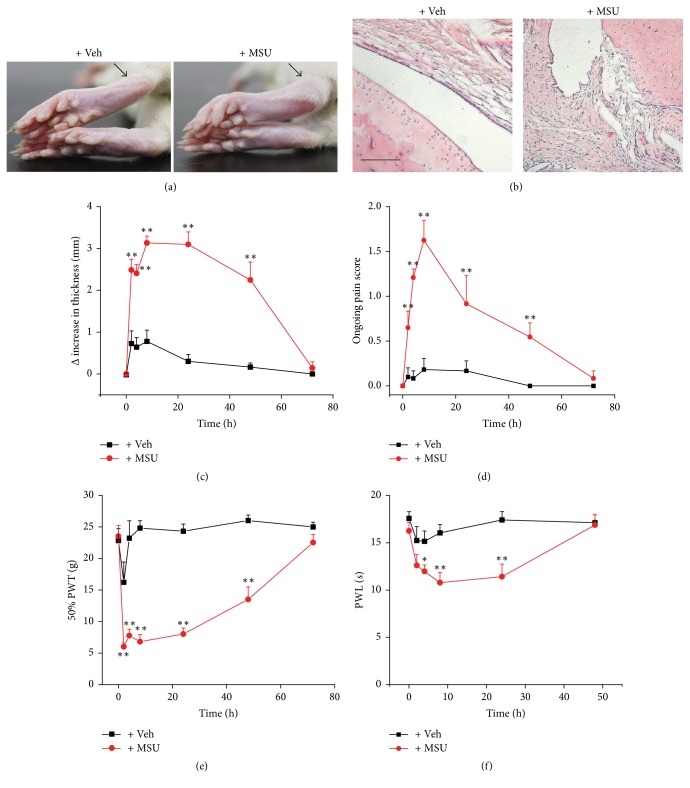
The establishment of a rat acute gout arthritis model and the characterization of the pain and inflammation properties. (a) Representative photos of rat ankle treated with either PBS (left panel, Veh group) or MSU (right panel, 1.25 mg/ankle, MSU group) via intra-articular (i.a.) injection. Black arrow indicates the injected ankle. (b) Representative microscopic photos of ankle synovial tissue sections from rats treated with vehicle or MSU. Whole sections are stained with hematoxylin/eosin. The scale bar indicates 50 *μ*m. (c–f) Time course of Δ increase of ankle thickness (c), ongoing pain score (d), 50% paw withdraw threshold (PWT, e), and paw withdraw latency (PWL, f) of Veh (in black) and MSU (in red) group rats. ^*∗*^*p* < 0.05 and ^*∗∗*^*p* < 0.01 versus Veh group. *n* = 6–8 rats/group.

Rats were injected with MSU (1.25 mg/ankle in 50 *μ*l PBS, i.a.) into the ankles to establish the gout model. Control group of rats were injected with vehicle (50 *μ*l PBS, i.a.). 8 h after MSU injection, MSU-treated rats showed obvious ankle swelling compared with vehicle-treated rats (Figure 1(a)). Microscopic evaluation of the histological sections of ankle synovial tissue revealed an extensive infiltration of inflammatory cells and focal synovial hyperplasia 8 h after MSU injection (Figure 1(b)). The ankle of MSU group rats showed extensive swelling, which appeared at 2 h, peaked at 8 h and lasted until 48 h after MSU injection (Figure 1(c)). The ankle swelling completely resolved 72 h after MSU injection. Moreover, MSU group rats showed overt ongoing pain behaviors, which included lifting, licking, and flinching the injected paw (Figure 1(d)). These behaviors lasted until 48 h after MSU injection (Figure 1(d)). The MSU group rats exhibited pronounced and long-lasting mechanical hypersensitivity, which peaked at 2 h and lasted until 48 h after MSU injection (Figure 1(e)). In addition, MSU group rats showed mild heat hypersensitivity, which developed at 4 h and lasted until 24 h after MSU injection (Figure 1(f)). Our data demonstrated that this rat gout model recapitulated the inflammation and pain response of human acute gouty attack. Therefore, our following experiments utilized this model and were conducted between 0 and 48 h after MSU injection to study whether and how EA attenuated MSU-induced acute gout arthritis.

### 3.2. Selection of Optimal EA Frequency for Alleviating Mechanical Hypersensitivity of the MSU-Induced Acute Gout Arthritis

**Figure 2:**
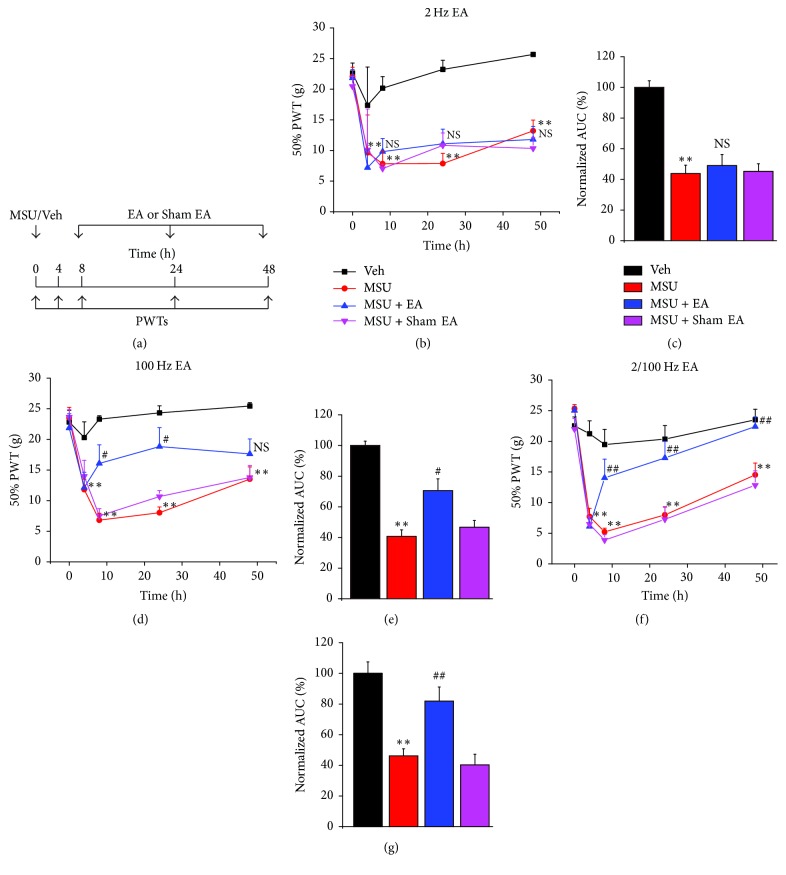
Evaluation of different frequencies of EA in treating mechanical hyperalgesia of MSU-induced acute gout arthritis. (a) Experimental scheme of EA or Sham EA treatment on MSU or Veh group rats. Rats were treated with saline (Veh) or MSU on 0 h to establish the gout model. PWT was quantified at 0, 4, 8, 24, and 48 h. EA or Sham EA was applied to MSU-treated rats 30 min before 8, 24, and 48 h observation time point. (b, d and f) Time courses of the effects of 2, 100, or 2/100 Hz EA on mechanical hyperalgesia of the rat gout model. (c, e, and g) Normalized area under the curve (AUC) of panels (b), (d), and (f) (from 0 to 48 h). AUCs were normalized to corresponding Veh group. ^*∗∗*^*p* < 0.01 versus Veh group. ^#^*p* < 0.05, ^##^*p* < 0.01 and NS: not significant versus MSU + Sham EA group. *n* = 6–10 rats/group.

It is important to determine an effective EA frequency on the pain response of MSU-induced acute gout arthritis at the beginning. Therefore, we chose three frequencies (2, 100, and 2/100 Hz) with fixed time duration (30 min) and pulse width (0.1 ms) in our following experiments according to previous literatures [20, 39]. EA or Sham EA was applied at bilateral “Zusanli” (ST36) and “Kunlun” (BL60) of gout model rats for 30 min for a total three times to study EA-induced antiallodynia on mechanical hypersensitivity (Figure 2(a)). As before, MSU injection into rats' ankle resulted in significant reduction of PWTs (Figure 2(b)). However, EA treatment with 2 Hz frequency did not elicit any obvious antiallodynia compared with Sham EA group at measured time points (Figure 2(b)). Area under the curve (AUC) of Figure 2(b) indicated no overall significant antiallodynia (Figure 2(c)). In contrast, EA treatment with 100 Hz frequency elicited significant antiallodynia compared with Sham EA group at 8 h and 24 h time points (Figure 2(d)). However, the third EA treatment failed to produce antiallodynia at 48 h time point, indicating a possible analgesic tolerance developed by repeated 100 Hz EA application (Figure 2(d)). AUC of Figure 2(d) indicated an overall antiallodynia produced by 100 Hz EA compared with Sham EA (Figure 2(e)). EA treatment with mixed 2/100 Hz frequency resulted in robust and long-lasting antiallodynia, which persisted until the third EA application (48 h time point) (Figure 2(f)). AUC of Figure 2(f) also indicated an obvious antiallodynia produced by repeated 2/100 Hz EA treatment compared with Sham EA (Figure 2(g)). The above results suggested that 2/100 Hz is the optimal EA frequency for alleviating mechanical hypersensitivity of MSU-induced acute gout arthritis compared with 2 and 100 Hz EA. Therefore, EA treatment with 2/100 Hz frequency was adopted in our following experiments.

### 3.3. Comparison of the Antiallodynia of EA and Indomethacin on Mechanical Hypersensitivity of MSU-Induced Acute Gout Arthritis

**Figure 3:**
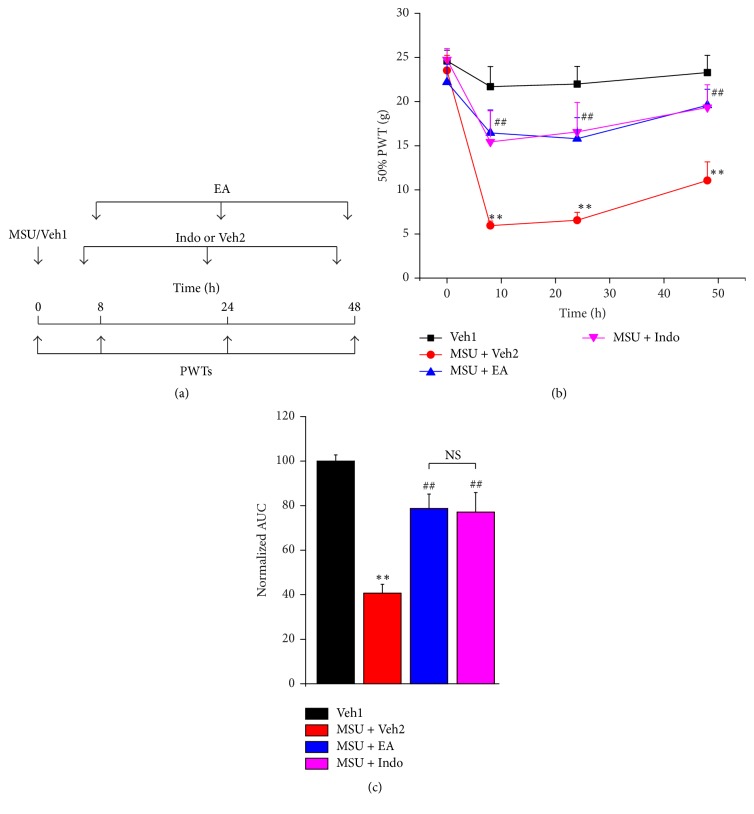
Comparison of the effect of EA and indomethacin on mechanical hyperalgesia of MSU-induced acute gout arthritis. (a) Experimental scheme of EA or indomethacin (Indo) treatment on rat gout model. Veh1 indicates PBS. Indomethacin (5 mg/kg) or vehicle (0.1% DMSO, Veh2) was injected (i.p.) to the rats 2 h and EA was applied 30 min before PWT quantification, respectively. (b) Time courses of the effects of 2/100 Hz EA and indomethacin on the mechanical hyperalgesia of rat gout model. (c) Normalized AUC of panel (b). ^*∗∗*^*p* < 0.01 versus Veh1 group. ^##^*p* < 0.01 versus MSU + Veh2 group. NS: not significant. *n* = 6 rats/group.

Next, we aimed to compare the antiallodynia of EA with indomethacin, the widely used NSAIDS for alleviating pain of gouty patients [40]. Indomethacin (5 mg/kg) or its vehicle (0.1% DMSO in PBS, Veh2) was administered (i.p.) to MSU rats 2 h before PWT measurement as indicated in Figure 3(a). Results in Figure 3(b) demonstrated that 2/100 Hz EA and indomethacin produced similar antiallodynia at all observed time points on mechanical hypersensitivity induced by MSU. AUC of Figure 3(b) indicated that the overall antiallodynia produced by 2/100 Hz EA and indomethacin are to similar extent (Figure 3(c)).

### 3.4. 2/100 Hz EA Reduced Ongoing Pain, Heat Hypersensitivity, and Ankle Swelling of MSU-Induced Acute Gout Arthritis

**Figure 4:**
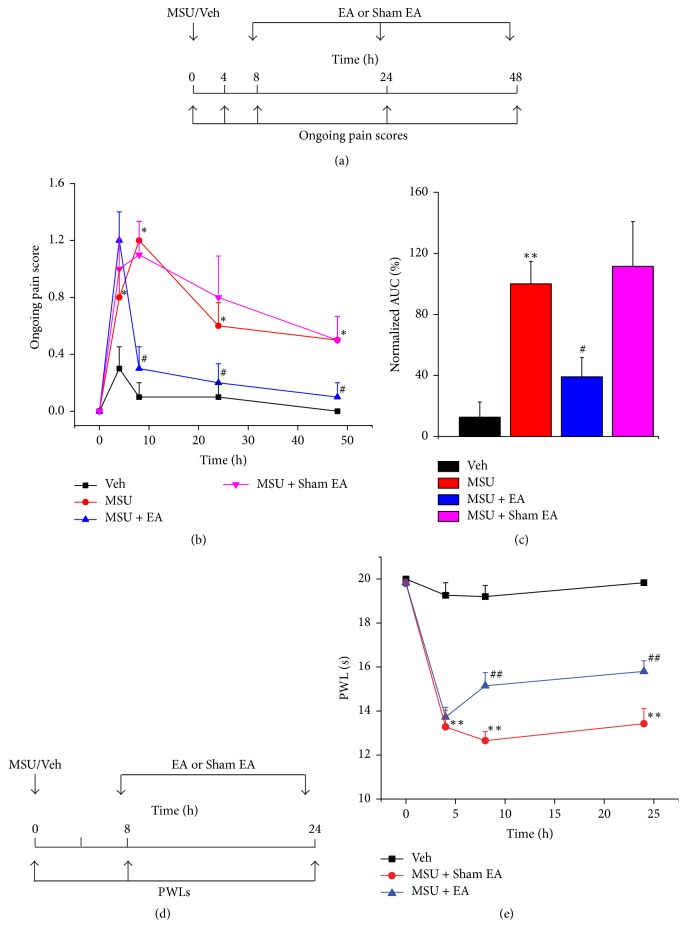
2/100 Hz EA attenuates ongoing pain score and heat hypersensitivity of MSU-induced acute gout arthritis. (a) Experimental scheme of 2/100 Hz EA or Sham EA treatment on ongoing pain score of rat gout model. (b) Time courses of the effects of 2/100 Hz EA on ongoing pain scores of the rat gout model. (c) Normalized area under the curve (AUC) of panel (b). ^*∗*^*p* < 0.05 and ^*∗∗*^*p* < 0.01 versus Veh group. ^#^*p* < 0.05 and ^##^*p* < 0.01 versus MSU + Sham EA group. *n* = 10 rats/group. (d) Experimental scheme of 2/100 Hz EA or Sham EA treatment on paw withdraw latency of rat gout model. (e) Time courses of the effects of 2/100 Hz EA or Sham EA on paw withdraw latency of rat gout model. ^*∗∗*^*p* < 0.01 versus Veh group. ^##^*p* < 0.01 versus MSU + Sham EA group. *n* = 6 rats/group.

We continued to study the therapeutic effects of 2/100 Hz EA on the ongoing pain behavior of MSU-induced acute gout arthritis. EA or Sham EA was applied and ongoing pain score was evaluated at time points as indicated in Figure 4(a). 2/100 EA treatment significantly alleviated the ongoing pain behavior of MSU-induced acute gout arthritis during all of the observation time points (Figure 4(b)). AUC of Figure 4(b) further indicated an overall antiallodynia of 2/100 Hz on the ongoing pain behavior compared with Sham EA group (Figure 4(c)). In addition, 2/100 Hz EA significantly reduced the heat hypersensitivity of MSU-induced acute gout arthritis (Figures 4(d) and 4(e)).

**Figure 5:**
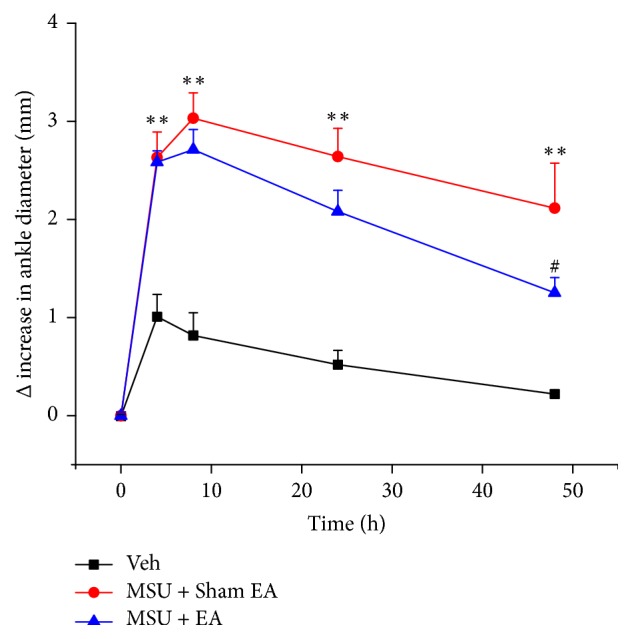
2/100 Hz EA reduced ankle swelling in MSU-induced acute gout arthritis. Time courses of the effect of 2/100 Hz EA or Sham EA on ankle diameter of MSU-induced acute gout arthritis rats. ^*∗∗*^*p* < 0.01 versus Veh group. ^#^*p* < 0.05 versus MSU + Sham EA group. *n* = 10 rats/group.

Based on the above persistent antiallodynia effect of 2/100 Hz EA, we proceeded to examine whether the signs of ankle inflammation induced by MSU could be attenuated by EA treatment as well. EA or Sham EA was applied as in Figure 4(a) and ankle diameter was measured 1 h after EA or Sham EA treatment. We found that EA significantly reduced the ankle diameter at 48 h time point compared with Sham EA. However, no significant difference of ankle diameter between EA and Sham EA was observed during 8 h and 24 h time points (Figure 5). In summary, the above results demonstrated that 2/100 Hz EA effectively reduced both pain and inflammation in MSU-induced acute gout arthritis in rats.

### 3.5. Involvement of Endogenous Opioid System in the Antiallodynia Effect Induced by 2/100 Hz EA

**Figure 6:**
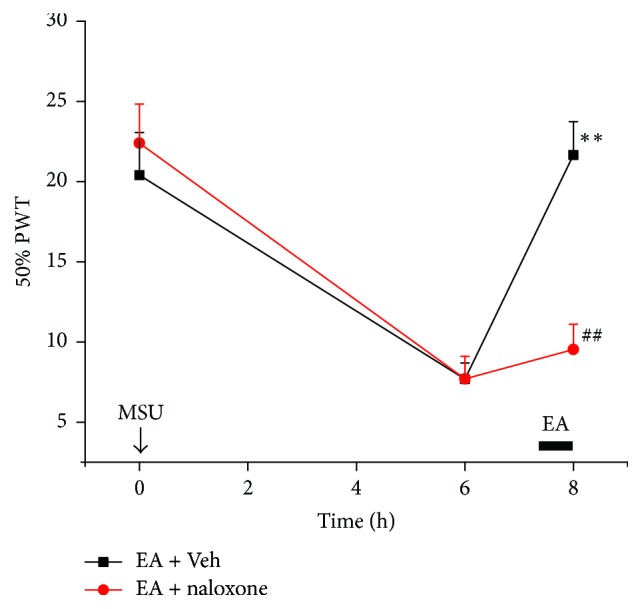
Effect of systematic administrations of naloxone on analgesic effects of EA on MSU-induced acute gout arthritis. Naloxone (2 mg/kg) or vehicle (PBS) was applied systematically to rats (i.p.) right before EA treatment. EA was applied to rats for 30 min as indicated and 50% PWT was evaluated as indicated. *n* = 6 rats/group. ^*∗∗*^*p* < 0.01 versus before EA treatment, ^##^*p* < 0.01 versus EA + Veh.

**Figure 7:**
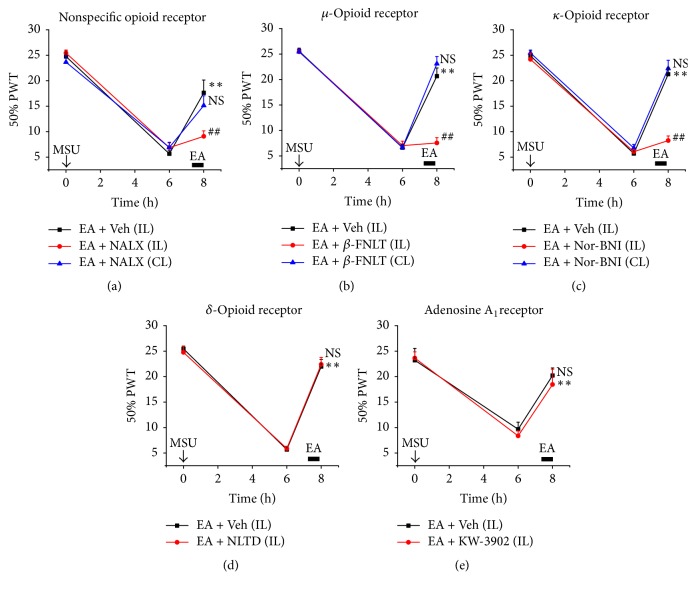
Effect of local administrations of opioid or adenosine receptor antagonists on analgesic effects of EA on MSU-induced acute gout arthritis. (a–e) Nonselective opioid receptor naloxone (NALX, 40 *μ*g/ankle), *μ*-opioid receptor antagonist *β*-funaltrexamine (*β*-FNLT, 50 *μ*g/ankle), *σ*-opioid receptor antagonist naltrindole (NLTD, 48.7 *μ*g/ankle), *κ*-opioid receptor antagonist nor-binaltorphimine (Nor-BNI, 76 *μ*g/ankle), or adenosine A1 receptor antagonist KW-3902 (600 *μ*g/ankle) was injected (i.a.) into the ankle either ipsi (IL) or contralaterally (CL) right before EA treatment. EA was applied to rats for 30 min as indicated. ^*∗∗*^*p* < 0.01 versus before EA treatment, ^##^*p* < 0.01 versus EA + Veh, NS: not significant versus EA + drug (IL) (panels (a)–(c)) or EA + Veh (IL) (panels (d) and (e)).

The involvement of endogenous opioids in 2/100 Hz EA-induced antiallodynia in MSU-induced acute gout arthritis was first tested by using naloxone, a nonselective opioid receptor antagonist. Naloxone (2 mg/kg, i.p.) was applied before EA treatment at 7.5 h time point. As shown in Figure 6, systematic naloxone pretreatment near completely reversed the antiallodynia induced by EA in MSU-induced acute gout arthritis. More recently, it is suggested that peripheral opioid system plays a crucial role in mediating EA-induced antiallodynia in certain inflammatory pain conditions [11, 12, 19]. Therefore, we proceeded to explore the involvement of peripheral opioid system in EA-induced antiallodynia on MSU-induced acute gout arthritis. Naloxone (40 *μ*g/ankle, i.a.) was locally administered to MSU-treated ankle before EA treatment at 7.5 h time point. As shown in Figure 7(a), locally injected naloxone significantly reduced the antiallodynia by EA. Moreover, when the same dosage of naloxone (40 *μ*g/ankle, i.a.) was injected into the contralateral, noninflamed ankle, the antiallodynia of EA on the ipsilateral ankle was unaffected (Figure 7(a)). Thus, the above results indicated that peripheral opioid receptors are involved in the antiallodynia of EA on MSU-induced acute gout arthritis.

To further explore the specific type of peripheral opioid receptors that were involved, we chose specific antagonists for *μ*-, *σ*-, and *κ*-opioid receptors and locally injected these antagonists to the ankle. Local injection of *μ*-receptor antagonist *β*-funaltrexamine (50 *μ*g/ankle) and *κ*-opioid receptor antagonist nor-binaltorphimine (76 *μ*g/ankle) but not *σ*-receptor antagonist naltrindole (48.7 *μ*g/ankle) significantly reversed the antiallodynia of EA on MSU-induced acute gout arthritis (Figures 7(b)–7(d)). Importantly, when the same dosages were injected into the contralateral, noninflamed ankle, *β*-funaltrexamine and nor-binaltorphimine did not affect the antiallodynia of EA on the ipsilateral inflamed ankle (Figures 7(b) and 7(c)). In addition, intraplanta injection of *β*-funaltrexamine, naltrindole, or nor-binaltorphimine at the same dosage into the hind paw of naïve rats did not produce any obvious effects on PWTs compared with control group (Table 2). We further tested whether peripheral adenosine A_1_ receptor was involved in the antiallodynia of EA on MSU-induced acute gout arthritis.  Figure 7(e) showed that local injection of adenosine A_1_ receptor antagonist KW-3902, at an effective dosage used previously (600 *μ*g/ankle) [12], did not affect EA-induced antiallodynia. In all, the above results indicated that peripheral *μ*- and *κ*-opioid receptors are involved in the antiallodynia of EA on MSU-induced acute gout arthritis.

### 3.6. 2/100 Hz EA Increased Expression of Endogenous Opioid Agonist in Local Tissue

**Figure 8:**
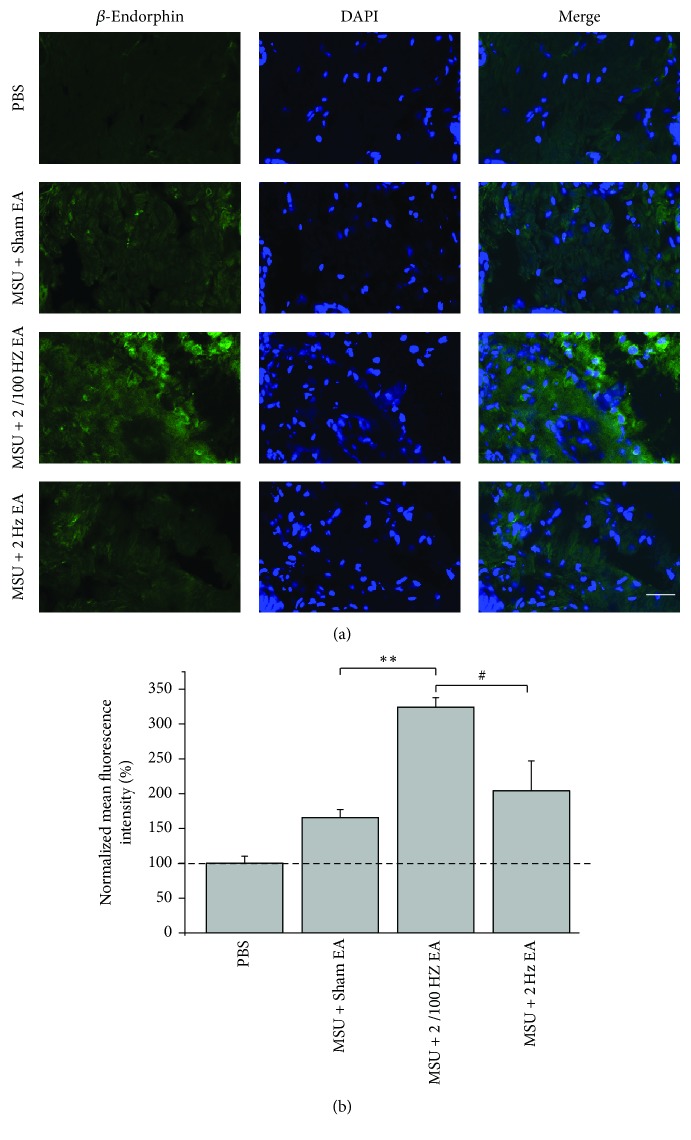
2/100 Hz EA increased expression of *β*-endorphin in local ankle skin tissues. (a) Representative immunofluorescence images of *β*-endorphin staining in ankle skin tissues isolated from vehicle (PBS), MSU + Sham EA, MSU + 2/100 Hz EA, and MSU + 2 Hz EA groups. Ankle skin tissues were isolated 30 min after EA or Sham EA treatment. Areas staining positive for *β*-endorphin were shown in green and nuclei were labeled with DAPI (blue). (b) Summary of the normalized mean fluorescence intensity (%) of *β*-endorphin staining as shown in (a). The mean fluorescence intensity of PBS group was taken as 100%; other groups were normalized thereafter. Scale bar indicates 20 *μ*m. *n* = 5-6 rats/group. ^*∗∗*^*p* < 0.01 and ^#^*p* < 0.05.

We further carried out immunohistochemistry study to explore whether 2/100 Hz EA can increase the expression of endogenous opioid agonist in local ankle tissue of acute gout arthritis rats. We found that 30 min 2/100 Hz EA treatment significantly increased the expression of *β*-endorphin in local ankle skin tissue isolated from MSU-treated rats (Figure 8(a)). Moreover, 2/100 Hz EA produced significantly more *β*-endorphin in local ankle skin tissue compared with 2 Hz EA (Figure 8(a)). The summarized data are shown in Figure 8(b).

### 3.7. Local Application of Opioid Receptor Agonists Produced Antiallodynia Effect on MSU-Induced Acute Gout Arthritis

**Figure 9:**
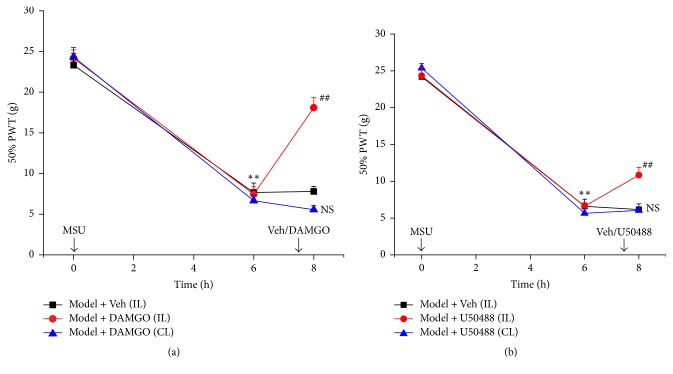
Effect of local administration of specific *μ*- and *κ*-opioid receptor agonists on mechanical hyperalgesia of MSU-induced acute gout arthritis rats. Opioid receptor agonists were locally administered to the ankle 7.5 h after MSU injection. PWTs were evaluated 30 min after opioid receptor application. (a-b) Effects of *μ*-receptor agonist DAMGO (4.9 *μ*g/ankle, panel (a)) and *κ*-receptor agonist (±) U50488 (1 *μ*g/ankle, panel (b)) injected (i.a.) into the ankle either ipsi (IL) or contralaterally (CL) on the mechanical hyperalgesia of MSU-induced acute gout arthritis rats. ^*∗∗*^*p* < 0.01 versus before MSU injection; ^##^*p* < 0.01 versus Model + Veh group. *n* = 6 rats/group.

We proceeded to investigate whether local injection of *μ*- and *κ*-opioid receptor agonists could produce antiallodynia in MSU-induced acute gout arthritis. Specific *μ*-receptor agonist DAMGO (4.9 *μ*g/ankle, i.a.) and *κ*-receptor agonist (±) U50488 (1 *μ*g/ankle, i.a.) were locally injected into the inflamed ankle of MSU-induced acute gout arthritis rats 30 min before PWT evaluation. As shown in Figures 8(a) and 8(b), DAMGO and (±) U50488 both produced significant analgesic effects on MSU-induced acute gout arthritis. Moreover, when injected into the contralateral, noninflamed ankle of MSU-induced acute gout arthritis rats, DAMGO and (±) U50488, at the same dosage used above, did not produce any signs of antiallodynia on the ipsilateral inflamed ankle (Figures 9(a) and 9(b)), indicating a local action of the used dosages. Therefore, the above results demonstrated that local application of *μ*- and *κ*-opioid receptor agonists could produce analgesic effect on mechanical hyperalgesia in MSU-induced acute gout arthritis rats, which mimics the effect of EA.

## 4. Discussion

In the present study, we established a rat model of acute gout arthritis by intra-articular injection of MSU into the ankle. By screening the optimal frequencies of EA, we found that 2/100 Hz EA produced the most robust and reliable analgesic effect on the mechanical hyperalgesia of the rat model of acute gout arthritis compared with 2 and 100 Hz. The analgesic effect of 2/100 Hz EA is comparable to that of the widely used NSAIDs indomethacin. Furthermore, 2/100 Hz EA also significantly alleviates the ongoing pain behavior, thermal hyperalgesia, and the ankle swelling of model rats. We further found that locally applied *μ*- and *κ*-opioid receptor antagonists significantly abolished the analgesic effect of 2/100 Hz EA, whereas locally applied *μ*- and *κ*-opioid receptor agonists produced significant analgesic effect, mimicking that of EA. Therefore, our study suggests that EA can effectively alleviate pain responses and inflammation in a rat model of acute gout arthritis and the analgesic effect of EA is mediated via peripheral *μ*- and *κ*-opioid receptors.

MSU deposition in and around the joint can cause leukocyte infiltration which engulfs MSU. This process will produce inflammatory and pronociceptive mediators. These mediators such as Il-1*β* and hydrogen peroxide can sensitize or directly activate nociceptors in the peripheral sensory nerve system and cause intense pain [5, 41–43]. We used an established rat model of acute gout arthritis by intra-articular injecting MSU into the ankle joint of rats. This model is a frequently used animal model for studying pain and inflammation mechanisms of gout. We found that the rats developed robust ankle swelling and pain response 2–4 h after MSU injection. At 8 h, the swelling and pain response reached peak. The ankle synovial tissue showed extensive infiltration of inflammation cells and hyperplasia. These signs are all in line with other studies using the same model and suggest the successful establishment of the gout model [27, 44]. But unlike other studies, we further extended our observation period and found that the ankle joint still exhibited obvious signs of pain and inflammation 48 h after MSU injection and subsided thereafter [27, 41, 44]. Therefore, the analgesic and anti-inflammatory effect of EA were examined between 8 and 48 h time window in our study.

By screening the optimal EA frequencies, we found that both high (100 Hz) and mixed frequency (2/100 Hz) EA produced analgesic effect on acute gout arthritis, whereas the effect of the latter is stronger and longer lasting. However, low-frequency (2 Hz) EA is not effective. The distinct effects produced by low, high, or mixed frequencies of EA have been reported in other studies as well. Similarly, 2/100 Hz or 100 Hz EA exerts sustained antinociception in a rat model of CFA-induced inflammatory pain, whereas 10 Hz EA is not effective at all [20]. It has also been demonstrated that 100 Hz but not 2 Hz EA produced preemptive analgesic effect against postincision pain. Conversely, low-frequency (2 or 10 Hz) EA produced more potent antiallodynic effect than high-frequency (100 Hz) in some neuropathic pain models such as diabetic neuropathic pain, oxaliplatin-induced neuropathic cold hypersensitivity, and paclitaxel-evoked peripheral neuropathy [45–47]. It is well known that low and high frequencies of EA can induce the release of different opioids from the central nerve system. We also found that 2/100 Hz EA produced more increase in endogenous opioid agonist *β*-endorphin in local inflamed ankle skin tissue compared with 2 Hz EA. Moreover, there are long-lasting differences in the activities of endogenous opioid system (including opioid release and opioid receptor expression) in the spinal cord and dorsal root ganglion between inflammatory and neuropathic pain conditions [37]. Therefore, these mechanisms may all underlie the distinct effectiveness of EA on different pain conditions. Therefore, our study, together with other findings mentioned above, suggests that optimal EA frequency needs to be selected beforehand in order to achieve the best analgesic effect on specific pain conditions.

In our study, we found that EA-induced analgesic effect was dramatically abolished by locally applied specific *μ*- and *κ*-opioid receptor antagonists. When the same doses of these antagonists were injected into the contralateral noninflamed ankle, EA-induced analgesic effect was not affected at all, suggesting that these dosages of antagonists only worked locally but not systematically. In addition, these antagonists, when given alone to the hind paw of naïve rats, did not modify basal pain threshold which rules out the possibility that these antagonists can cause pain per se. These data all suggest that EA-induced analgesic effect on acute gout arthritis is largely mediated via peripheral opioid receptors. This conclusion is further corroborated by our findings that locally applied specific *μ*- and *κ*-opioid receptor agonists produced analgesic effect in the acute gout arthritis model, mimicking the analgesic effect of EA. In addition to the well-established central opioid system, peripheral opioid system has also been suggested to play an important role in EA-induced antiallodynia. It has been reported that local application of opioid receptor antagonists significantly abolished EA-induced analgesia in CFA or carrageenan-induced inflammatory pain [20–22]. Local injection of antibodies against opioid peptides enkephalin or endomorphin significantly inhibited EA-mediated mechanical and thermal antinociception [19]. Two more recent studies also demonstrated that application of naloxone methiodide, a peripherally acting nonselective opioid antagonist, almost totally abolished EA-induced analgesia in CFA-induced inflammatory pain and acid-induced fibromyalgia models, suggesting the involvement of peripheral opioid system in EA-mediated analgesic effect [11, 12]. It is now well established that opioid receptors are expressed in peripheral sensory neurons in addition to central nervous system [48]. EA can promote the migration of opioid peptide-containing monocytes/macrophages to the local inflamed site and release opioid peptides locally [19]. Opioid peptides bind to opioid receptors on the peripheral sensory neurons and exert peripheral analgesia effect [19, 49]. In our study, we further observed that EA significantly upregulated the expression of endogenous opioid receptor agonist *β*-endorphin in local ankle skin tissue. Although we cannot totally rule out the participation of central opioid system in the present study, our present results convincingly suggest that peripheral opioid systems are involved in EA-induced analgesic effect on acute gout arthritis.

In addition to the analgesic effect, EA also showed anti-inflammatory effect on the rat model of acute gout arthritis. This observation is in line with several other studies showing that EA elicits anti-inflammatory effects on some animal inflammatory pain models [9, 20, 50–52]. However, we observed that the onset of the anti-inflammatory effect of EA appeared much later than its analgesic effect. It was not until 48 h time point that EA showed significant anti-inflammatory effect. This is in sharp contrast to EA's analgesic effect, which took place right after EA application. This result is consistent with our previous findings that EA-induced antiallodynia is not synchronized with its anti-inflammatory effect in CFA-induced inflammatory pain model [9]. The difference in the time frame of these two events leads us to postulate that the antiallodynia effect of EA is independent of its anti-inflammatory effect, at least in the initial stage of antiallodynia (from 8 to 24 h). The underlying mechanisms of EA-induced anti-inflammatory effect still need to be further investigated.

## 5. Conclusions

Our study demonstrated that 2/100 Hz EA effectively alleviates pain response and inflammation in a rat model of acute gout arthritis. EA can produce equivalent analgesic effect compared with the widely used NASIDs indomethacin. The analgesic effect of EA on acute gout arthritis is largely mediated via peripheral *μ*- and *κ*-opioid receptors. These results suggest that EA may be used as a potential complementary therapeutic option for treating acute gout arthritis.

## Figures and Tables

**Table 1:** Summary of the drugs used in the present study and the citations for their usage.

Action	Target	Name	Abbreviation	*In vivo* dosage	Reference
Antagonist	Opioid receptors	Naloxone	NALX	2 mg/kg (i.p.) 40 *µ*g/ankle (i.a.)	[31]
	*µ*-Opioid receptor	*β*-Funaltrexamine	*β*-FNLT	50 *µ*g/ankle (i.a.)	[32]
	*κ*-Opioid receptor	Nor-binaltorphimine	Nor-BNI	76 *µ*g/ankle (i.a.)	[32]
	*σ*-Opioid receptor	Naltrindole	NLTD	48.7 *µ*g/ankle (i.a.)	[32]
	A_1_ receptor COX	KW-3902 Indomethacin	— Indo	600 *µ*g/ankle (i.a.) 5 mg/kg (i.p.)	[12] [35]

Agonist	*µ*-Opioid receptor	DAMGO	—	4.9 *µ*g/ankle (i.a.)	[34]
	*κ*-Opioid receptor	(±) U50488	—	1 *µ*g/ankle (i.a.)	[33]

**Table 2:** The effects of the opioid receptor antagonists on 50% paw withdraw threshold (PWT) of naïve rats. Opioid receptor antagonists and corresponding vehicle (PBS, control) were administered intraplantarly into the hind paws of naïve rats. 50% PWTs were measured before and after drug/vehicle treatment. NS: not significant compared with control.

Antagonists (*µ*g)	50% PWT (g)	
	Baseline	Treated
Control	25.4 ± 0.6	23.3 ± 1.9
Naloxone (40)	24.3 ± 1.1	21.8 ± 2.1 (NS)
*β*-Funaltrexamine (50)	24.8 ± 1.2	25.4 ± 0.6 (NS)
Nor-binaltorphimine (76)	25.4 ± 0.6	24.3 ± 1.1 (NS)
Naltrindole (48.7)	25.4 ± 0.6	24.9 ± 0.7 (NS)
